# Macrophage peroxisome proliferator-activated receptor *γ* deficiency delays skin wound healing through impairing apoptotic cell clearance in mice

**DOI:** 10.1038/cddis.2014.544

**Published:** 2015-01-15

**Authors:** H Chen, R Shi, B Luo, X Yang, L Qiu, J Xiong, M Jiang, Y Liu, Z Zhang, Y Wu

**Affiliations:** 1 Department of Basic Medicine, Institute of Immunology, Third Military Medical University of PLA, Chongqing, China

## Abstract

Skin wound macrophages are key regulators of skin repair and their dysfunction causes chronic, non-healing skin wounds. Peroxisome proliferator-activated receptor gamma (PPAR*γ*) regulates pleiotropic functions of macrophages, but its contribution in skin wound healing is poorly defined. We observed that macrophage PPAR*γ* expression was upregulated during skin wound healing. Furthermore, macrophage PPAR*γ* deficiency (*PPARγ-*knock out (KO)) mice exhibited impaired skin wound healing with reduced collagen deposition, angiogenesis and granulation formation. The tumor necrosis factor alpha (TNF-*α*) expression in wounds of *PPARγ-*KO mice was significantly increased and local restoration of TNF-*α* reversed the healing deficit in *PPARγ*-KO mice. Wound macrophages produced higher levels of TNF-*α* in *PPARγ*-KO mice compared with control. *In vitro*, the higher production of TNF-*α* by *PPARγ*-KO macrophages was associated with impaired apoptotic cell clearance. Correspondingly, increased apoptotic cell accumulation was found in skin wound of *PPARγ-*KO mice. Mechanically, peritoneal and skin wound macrophages expressed lower levels of various phagocytosis-related molecules. In addition, PPAR*γ* agonist accelerated wound healing and reduced local TNF-*α* expression and wound apoptotic cells accumulation in wild type but not *PPARγ*-KO mice. Therefore, PPAR*γ* has a pivotal role in controlling wound macrophage clearance of apoptotic cells to ensure efficient skin wound healing, suggesting a potential new therapeutic target for skin wound healing.

The skin is the largest human organ that is essential to protect body against infection and excessive water loss. However, skin is also very easily injured by various attacks.^1^ After injury, the skin needs to restore homeostasis, structure integrity and functional competence.^2^ Skin wound healing is a complicated process orchestrated by interactions of inflammatory cells, resident cells, extracellular matrix components and soluble mediators. The healing process is usually divided into three sequential and overlapping phases: inflammation, proliferation and maturation.^3^ The inflammatory phase includes platelet aggregation, blood coagulation and inflammatory cells recruitment to wound sites. The proliferative phase involves keratinocytes, fibroblasts and endothelial cells migration and proliferation, contributing to reepithelialization, collagen deposition and angiogenesis. And the maturation phase restores tissue structure integrity and functional competence.^2^ If wounds do not progress in the timely and orderly manner, they convert into chronic, non-healing wounds that are a growing world health-care problem related with increasing incidence of diabetes, obesity and aging.^4, 5, 6^

Macrophages are the most important immune cells recruited to the wound sites following skin injury, which exhibit pleiotropic functions to orchestrate the healing process throughout the different phases.^1, 7, 8^ During the earlier inflammation phase, macrophages characterize an pro-inflammatory phenotype, they release pro-inflammatory mediators such as tumor necrosis factor alpha (TNF-*α*), nitric oxide and IL-6, and produce protease and reactive oxygen species to combat contaminating organisms. In pathogen spread wounds, macrophages phagocytose these pathogens and present antigen to T cells. Macrophages also phagocytose wound debris and apoptotic cells.^9^ Phagocytosis of apoptotic cells predominately switches pro-inflammatory macrophages to anti-inflammatory/wound-healing macrophages to resolve wound inflammation and initiate the healing process.^10, 11^ During the later healing phase, macrophages characterize anti-inflammatory/wound-healing phenotype, they produce many cytokines, chemokines and growth factors to crosstalk with keratinocytes, fibroblasts and endothelial cells, contributing to reepithelialization, collagen deposition, angiogenesis, granulation tissue formation and wound repair.^12^ In wound healing, non-functional or dysfunctional macrophages are related with chronic, non-healing wounds.^7, 13, 14, 15^ Therefore, sustaining macrophage normal functions is critical for successful wound healing. However, the underlying factors regulate macrophage functions during wound healing are not fully known.

Peroxisome proliferator-activated receptors (PPARs) are ligand-activated transcription factors belonging to the nuclear receptor superfamily, which includes three isotypes, PPAR*α*, PPAR*β*/*δ* and PPAR*γ*. In skin wound healing, PPAR*α* participates in the control of the early inflammation phase of the healing,^16^ PPAR*β* regulates keratinocytes proliferation, adhesion and migration;^16, 17, 18^ and PPAR*δ* promotes fibroblast proliferation.^19^ However, the role of PPAR*γ* in wound healing is not elucidated. It is well known that PPAR*γ* is a key factor transcriptionally coordinates macrophage functions.^20^ Macrophage PPAR*γ* signaling is essential for the efficient clearance of apoptotic cells^21, 22^ and the switching from pro-inflammatory macrophages to anti-inflammatory macrophages,^23, 24^ which are important for resolving inflammation and maintaining homeostasis. In this study, we generated mice with macrophage PPAR*γ* deficiency to investigate the role of macrophage PPAR*γ* in the healing of skin wounds.

## Results

### PPAR*γ* is upexpressed in wounded skin and wound macrophage

**Figure 1:**
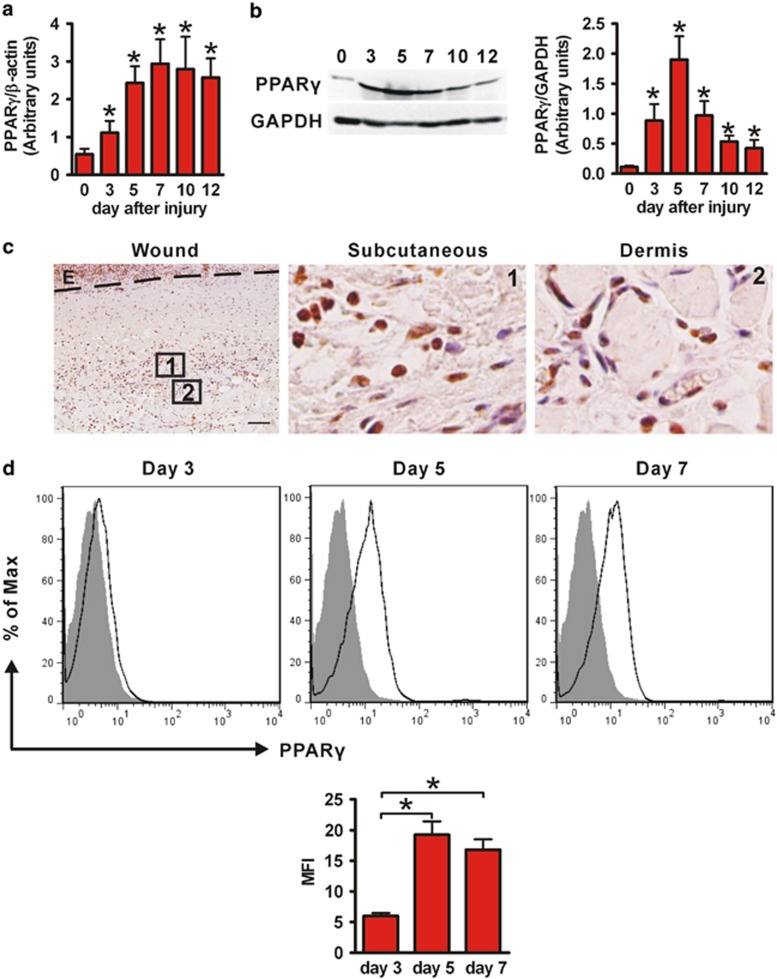
PPAR*γ* expression during wound healing of WT mice. (**a**) mRNA and (**b**) protein levels of PPAR*γ* in wounds. mRNA expression (**a**) is normalized to *β*-actin and the protein expression (**b**) is normalized to GAPDH. *P<0.05, wounded skin *versus* normal skin. (**c**) Immunohistochemical staining of PPAR*γ* expression in wounded skin on day 5 after wounding. Boxed areas of s.c. (number 1) and dermis (number 2) tissue in the left panel are enlarged in the middle and right panel. Black hatched line lines eschar. E, eschar. Scale bar=50 *μ*m. (**d**) Flow cytometric analysis of PPAR*γ* expression in wound macrophages on days 3, 5 and 7. CD11b^+^F4/80^+^ was used to gate macrophages. Isotype control, gray histogram; PPAR*γ*, unshaded histogram. Mean fluorescence intensity (MFI) is shown. *P<0.05. Data are expressed as mean±S.D. and images are representative, *n*=3 for each time point

We first investigated the temporal and spatial expression of PPAR*γ* during skin wound healing in wild-type (WT) mice (Figures 1a and b). Low levels of PPAR*γ* (mRNA and protein) were observed in unwounded control skin (day 0). However, a significant increase of mRNA and protein levels of PPAR*γ* was observed after wounding (days 3, 5, 7, 10 and 12). Immunohistochemical staining showed that PPAR*γ* protein was significantly enhanced in both subcutaneous (s.c.) and dermis of wounded skin (Figure 1c) compared with normal skin (Supplementary Figure 1). In addition, flow cytometric analysis showed that wound macrophage upregulated PPAR*γ* expression during the healing process (Figure 1d). These results suggest a potential involvement of macrophage PPAR*γ* in the regulation of skin wound healing.

### Characterization of macrophage PPAR*γ* deficiency mice

To investigate the role of macrophage PPAR*γ* in wound healing, conditional knock out (KO) mice lacking macrophage expression of PPAR*γ* were generated by crossing mice bearing the lox-P-targeted *PPARγ* (*PPARγ*^*f/f*^) allele with mice bearing the *lysozyme-M Cre* (*LysMCre*) recombinase transgene. We refer to *PPARγ*^*f/f*^*LysMCre*^*−*^ mice as control (*PPARγ*-WT), and their *PPARγ*^*f/f*^*LysMCre*^*+*^ littermates as KO animals (*PPARγ*-KO).

**Figure 2:**
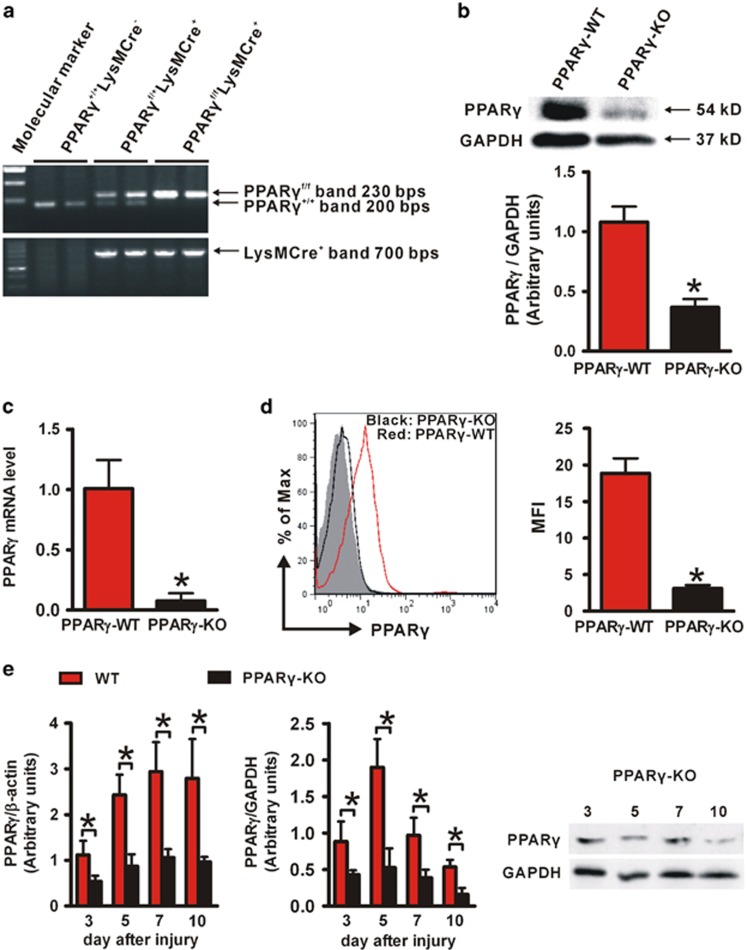
Characterization of macrophage-specific PPAR*γ* deficiency mice. (**a**) Genotyping analysis of *PPARγ*^*f/f*^*LysMCre*^*+*^, *PPARγ*^*f/+*^*LysMCre*^*+*^ and *PPARγ*^*+/+*^*LysMCre*^*−*^ (*PPARγ*-WT) mice. (**b**) WB and (**c**) RT-PCR analysis of PPAR*γ* expression in isolated peritoneal *PPARγ*-WT and *PPARγ*-KO macrophages. Protein expression is normalized by GAPDH. mRNA expression is normalized by *β*-actin and represented as the fold change in *PPARγ*-KO macrophages compared with *PPARγ*-WT macrophages. (**d**) Flow cytometric analysis of PPAR*γ* expression in isolated *PPARγ*-WT and *PPARγ*-KO wound macrophages. CD11b^+^F4/80^+^ was used to gate macrophages. Isotype control, gray histogram; PPAR*γ*, unshaded histogram (red histogram: *PPARγ*-WT; black histogram: *PPARγ*-KO). Mean fluorescence intensity (MFI) is shown. For (**b**–**d**), *P<0.05, *PPARγ*-KO *versus PPARγ*-WT. (**e**) mRNA and protein levels of PPAR*γ* in wounds of WT and *PPARγ*-KO mice. mRNA expression is normalized by *β*-actin and protein expression is normalized by GAPDH. *P<0.05. Data are expressed as mean±S.D. and images are representative, *n*=3 for each time point and group

Genotyping of the tail DNA confirmed the presence of *Cre* transgene in *PPARγ*^*f/+*^*LysMCre*^*+*^ and *PPARγ*^*f/f*^*LysMCre*^*+*^ mice and its absence in WT mice (Figure 2a). Peritoneal macrophages from *PPARγ*-KO mice showed significant lower levels of PPAR*γ* mRNA and protein compared with their control macrophages (Figures 2b and c), and wound macrophages from *PPARγ*-KO mice had lower PPAR*γ* expression (Figure 2d). In addition, both *PPARγ*-WT and *PPARγ*-KO wound neutrophils showed no evidence for PPAR*γ* staining, and PPAR*γ* expression in splenic T cells, B cells and dendritic cells were not significantly different between *PPARγ*-WT and *PPARγ*-KO mice (Supplementary Figure 2). These results indicated an efficient and specific macrophage PPAR*γ* ablation.

In addition, the temporal profile of PPAR*γ* mRNA and protein levels in skin wounds were compared between *PPARγ*-KO and *PPARγ*-WT mice. Significant lower levels of PPAR*γ* (mRNA and protein) were observed in *PPARγ*-KO mice compared with WT mice after wounding (days 3, 5, 7 and 10; Figure 2e), indicating an important contribution of macrophage PPAR*γ* to the increased PPAR*γ* expression observed during skin wound healing.

### Delayed wound healing in mice with macrophage PPAR*γ* deficiency

**Figure 3:**
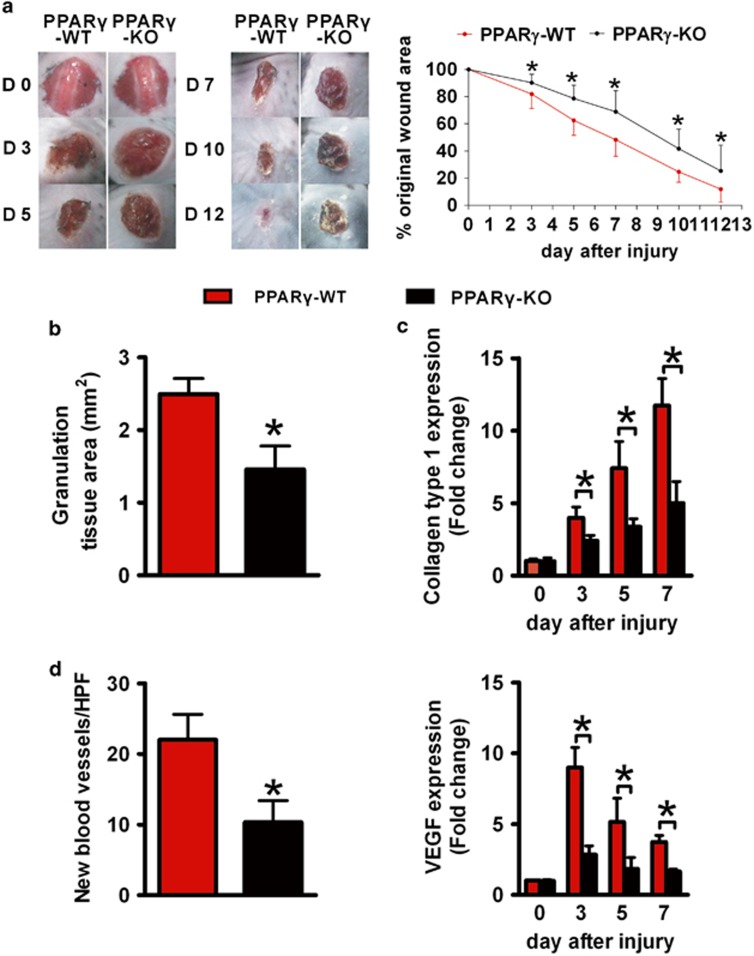
Delayed wound healing in *PPARγ*-KO mice. (**a**) Representative wounds, and statistical analysis of wound areas expressed as percentage of the initial (day 0) wound size. *n*=12, for each time point and group. (**b**) The areas of granulation tissue in 5-day-old wounds. (**c**) mRNA expression of collagen type 1 in wounds. (**d**) Quantification of new blood vessels in the granulation tissue of 5-day-old wounds in 3–5 high-power fields (HPFs) per mice, and mRNA expression of VEGF in wounds. For (**b**–**d**), *n*=3 for each time point and group. All data are expressed as mean±S.D. *P<0.05, *PPARγ*-WT *versus PPARγ*-KO mice

Thereafter, full-thickness circular wounds were produced on *PPARγ*-WT and *PPARγ*-KO mice. The wound sizes were monitored daily. In addition, we found that wound closure in *PPARγ*-KO mice was significantly delayed from 3 to 12 after wounding compared with *PPARγ*-WT mice (Figure 3a). Next, we detected granulation formation, collagen deposition and angiogenesis during wound healing, focusing on days 5 and 7, when mainly granulation tissue formation, collagen deposition and angiogenesis occur.

Hematoxylin and eosin (H&E) staining of 5-day-old wounds showed that areas of granulation tissue was markedly reduced at the wound sites in *PPARγ*-KO mice compared with *PPARγ*-WT mice (Figure 3b and Supplementary Figure 3a).

To examine collagen deposition, 5- and 7-day-old wounds were stained with Masson trichrome. At the wound edge, characterized by the acanthotic keratinocyte layer, new and old collagen can be observed. The old collagen away from the wound margin in both strains of mice stains dark blue (Supplementary Figure 3b, closed arrow). The newly formed collagen near the wound margin is below the acanthotic keratinocyte layer (Supplementary Figure 3b, open arrow). Both 5- and 7-day-old wounds in *PPARγ*-KO mice contained lower areas of new collagen compared with the *PPARγ*-WT wounds (Supplementary Figure 3b). To further quantitatively assess collagen deposition, collagen type 1 mRNA expression was analyzed by real-time polymerase chain reaction (RT-PCR) and a significantly higher level was detected on days 3, 5 and 7 wounds of *PPARγ*-WT mice compared with that of *PPARγ*-KO mice (Figure 3c).

To examine the rate of angiogenesis, immunohistochemistry analysis of CD31 was carried out. The wounds of *PPARγ*-WT had higher density of new blood vessels in the granulation tissue compared with wounds of *PPARγ*-KO mice (Figure 3d and Supplementary Figure 3c). Consistently, the mRNA level of vascular endothelial growth factor (VEGF) in wounds was significantly decreased in *PPARγ*-KO mice compared with *PPARγ*-WT mice (Figure 3d).

Overall, wound healing is severely impaired both macroscopically and microscopically in *PPARγ*-KO mice.

### Wounds of *PPARγ*-KO mice exhibit normal numbers of neutrophils and macrophages

**Figure 4:**
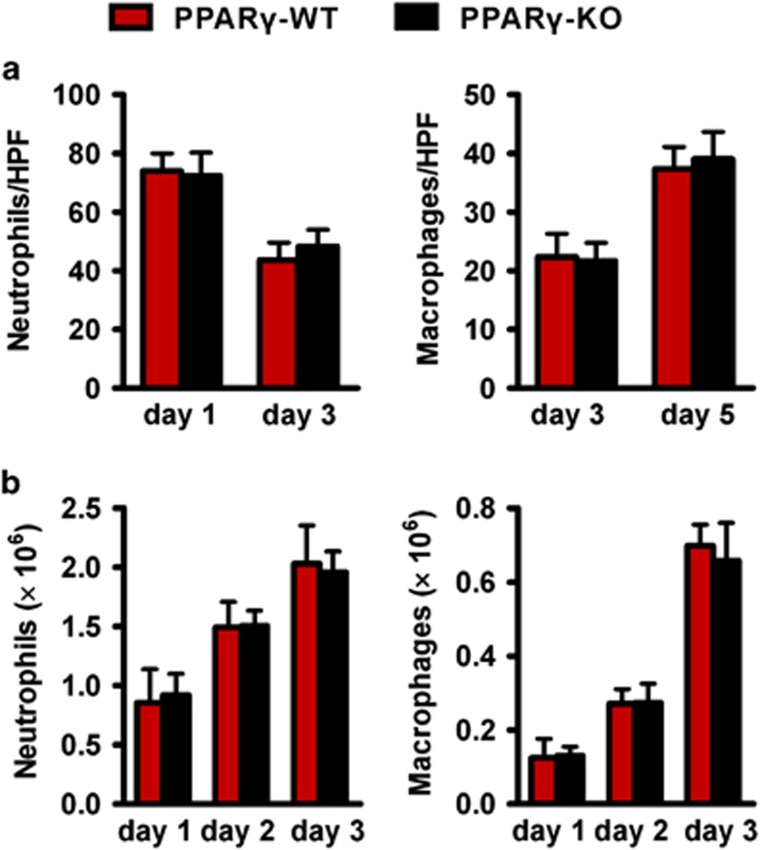
There is no difference in inflammatory cell recruitment to the wounds of *PPARγ*-WT and *PPARγ*-KO mice. (**a**) Quantification of Ly-6G^+^ neutrophils within granulation tissues of 1- and 3-day-old wounds, and F4/80^+^ macrophages within granulation tissues in 3- and 5-day-old wounds in 3–5 high-power fields (HPFs) per mice. (**b**) Wound cells derived from PVA sponges after days 1, 2 and 3 inserting. The numbers of neutrophils and macrophages were shown. SSC^high^Ly-6G^+^ was used to gate neutrophils and FSC^high^F4/80^+^ was used to gate macrophages. Data are expressed as mean±S.D., *n*=3 for each time point and group

It is well known that inflammatory cells have a vital role in normal skin wound healing and impairing inflammatory cell recruitment to wound sites severely affects wound healing.^25, 26, 27, 28, 29, 30, 31^ So we next measured the leukocyte accumulation to skin wound sites between *PPARγ*-WT and *PPARγ*-KO mice. Immunohistochemistry staining of day 3 wounds with antibodies specific to neutrophils (Ly-6G) or macrophages (F4/80) were shown (Supplementary Figure 4). The numbers of neutrophils and macrophages were counted within the granulation tissues of wounds at indicated time points and no significant difference between both mice strains was seen (Figure 4a). Furthermore, flow cytometry was used to measure the numbers of neutrophils and macrophages in skin wound on days 1, 2 and 3 after polyvinyl alcohol (PVA) sponge insertion, and no significant differences were observed between both mice strains as well (Figure 4b). These results indicated that the recruitment of neutrophils and macrophages to skin wound in *PPARγ*-WT and *PPARγ*-KO mice are similar.

### Local restoration of TNF-*α* rescues impaired wound healing in *PPARγ*-KO mice

**Figure 5:**
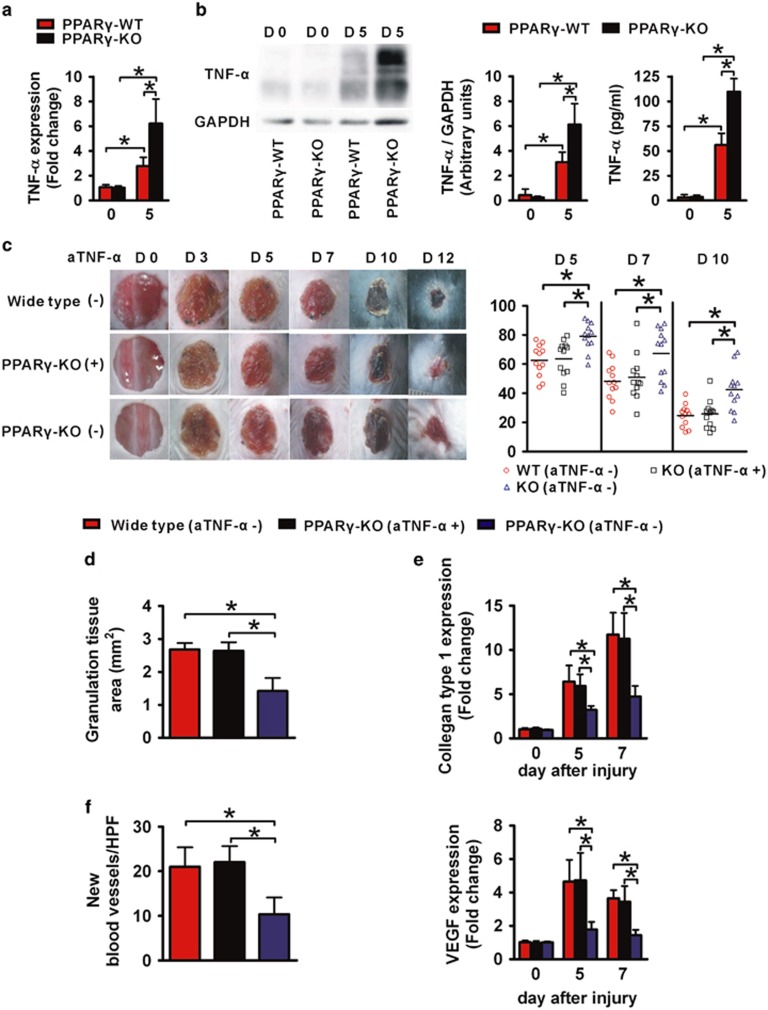
Enhanced expression of TNF-*α* in wounds is causal for the wound healing defect of *PPARγ*-KO mice. (**a**) mRNA and (**b**) protein expression of TNF-*α* in normal skin and 5-day-old wounds. (**c**) Representative wounds, and statistical analysis of wound areas expressed as percentage of the initial (day 0) wound size. *n*=12, for each time point and group. (**d**) The areas of granulation tissue in 5-day-old wounds. (**e**) mRNA expression of collagen type 1 in wounds. (**f**) Quantification of new blood vessels in the granulation tissue of 5-day-old wounds in 3–5 high-power fields (HPFs) per mice, and mRNA expression of VEGF in wounds. For (**a**), (**b**), (**d**), (**e**) and (**f**), *n*=3 for each time point and group. All data are expressed as mean±S.D. and images are representative. *P<0.05

Other than inflammatory cells, cytokines, growth factors and chemokines regulate the wound healing as well. Accumulated evidences indicate that enhancing local TNF-*α* expression delays wound healing, reduces collagen deposition and suppresses angiogenesis.^32, 33, 34, 35, 36, 37^ So we next measured TNF-*α* levels in wound tissues of *PPARγ*-KO *versus PPARγ*-WT mice by RT-PCR, western blotting (WB) and ELISA. Neither mRNA levels nor protein expression of TNF-*α* have significant difference between *PPARγ*-KO and *PPARγ*-WT mice in day 0 wounds (Figures 5a and b). After injury, the expression levels of TNF-*α* were increased in 5-day-old wounds of both strains mice. However, a significant higher expression of TNF-*α* both at mRNA and protein level were observed in 5-day-old wounds of *PPARγ*-KO mice compared with *PPARγ*-WT mice (Figures 5a and b).

These results suggested a significant increase of TNF-*α* following macrophage PPAR*γ* deficiency, which may account for the delayed wound healing. So we next s.c. injected rat anti-mouse TNF-*α* (aTNF-*α*) around the wound.^35, 36^ In addition, mock injections of isotype control antibody were taken as controls. In *PPARγ*-KO mice, local injection of aTNF-*α* improved skin wound healing, granulation tissue formation, collagen deposition and angiogenesis to the level similar to that in WT mice without any treatment, indicating a full rescue (Figures 5c–f and Supplementary Figures 5a and c). These data suggest that the increased local TNF-*α* expression is causal to the delayed wound healing in *PPARγ*-KO mice.

### Impaired apoptotic cell clearance by macrophages contributes to increased wound TNF-*α* expression in *PPARγ*-KO mice

**Figure 6:**
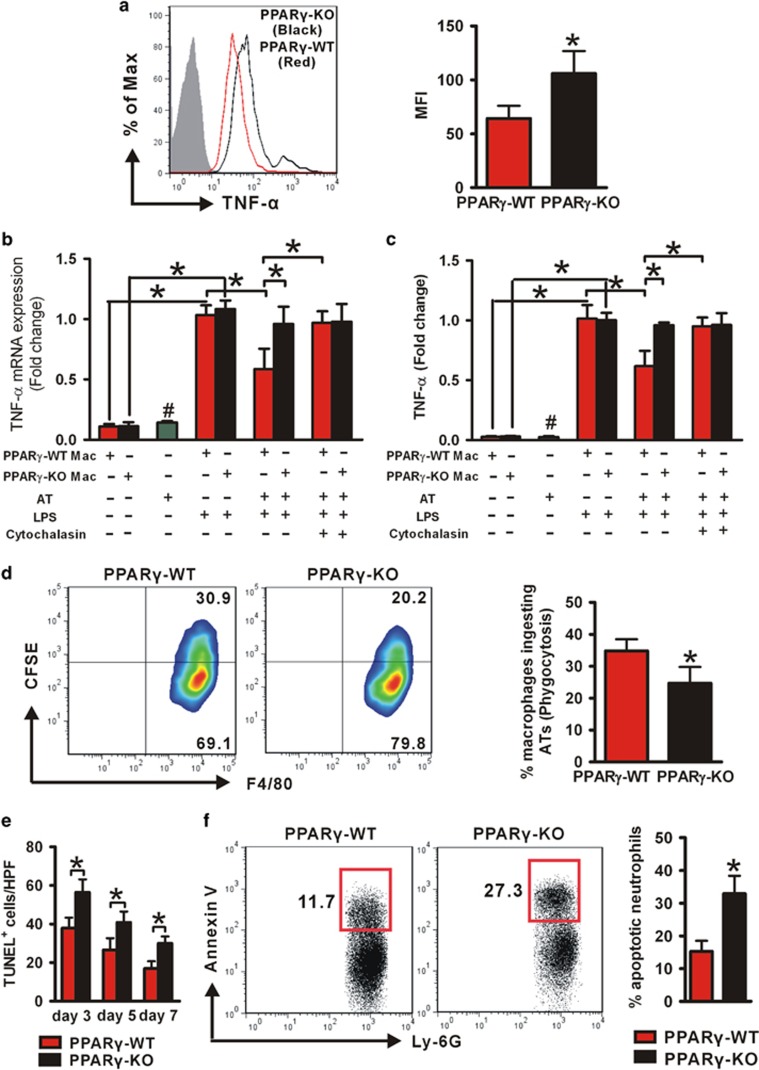
Increased production of TNF-*α* is due to impaired macrophage phagocytosis in *PPARγ*-KO mice. (**a**) Intracellular staining for TNF-*α* in wound macrophages derived from PVA sponges after 5 days implantation. F4/80^+^ was used to gate macrophages. Isotype control, gray histogram; TNF-*α*, unshaded histogram (red histogram: *PPARγ*-WT; black histogram: *PPARγ*-KO). Mean fluorescence intensity (MFI) is shown. The mRNA (**b**) and protein (**c**) levels of TNF-*α* in *PPARγ*-WT and *PPARγ*-KO peritoneal macrophages, and in ATs. *P<0.05. ^#^*P*<0.05, ATs *versus PPARγ*-WT or *PPARγ*-KO macrophages stimulated by LPS. (**d**) Representative flow cytometric raw data of the phagocytosis assays (macrophages were stained using F4/80 PE Ab; ATs were loaded with CFSE), and the percentages of macrophages ingesting ATs. (**e**) Quantification of TUNEL^+^ cells in 3-, 5- and 7-day-old wounds in 3–5 high-power fields (HPFs) per mice. (**f**) The percentages of apoptotic neutrophils in total wound cells derived from PVA sponges after day 3 inserting. Data are expressed as mean±S.D. and images are representative, *n*=3 for each time point and group. For (**a**), (**d**), (**e**) and (**f**), *P<0.05, *PPARγ*-WT *versus PPARγ*-KO mice

As macrophages represent a major source of the cytokines in wounds,^38^ we speculated that *PPARγ*-KO macrophages may release more TNF-*α* compared with *PPARγ*-WT macrophages during wound healing. Therefore, we detected TNF-*α* expression in isolated wound macrophages and found that *PPARγ*-KO wound macrophages expressed more TNF-*α* than their *PPARγ*-WT counterparts (Figure 6a).

To explore how PPAR*γ* regulates wound macrophage TNF-*α* production, we next analyzed TNF-*α* mRNA levels in peritoneal macrophages of both mice strains stimulated with or without lipopolysaccharide (LPS). Unstimulated *PPARγ*-WT and *PPARγ*-KO macrophages expressed similar low-level of TNF-*α* (Figure 6b). Although LPS greatly upregulated TNF-*α* expression in *PPARγ*-WT and *PPARγ*-KO macrophages, no significant difference was observed between these two stains of macrophages (Figure 6b). In addition, similar results were further confirmed at protein level (Figure 6c), indicating that PPAR*γ* has no direct effect on TNF-*α* production in macrophages after LPS stimulation. However, in the presence of apoptotic thymocytes (ATs), *PPARγ*-WT macrophages significantly reduced LPS-induced TNF-*α* production (at both mRNA and protein level), but *PPARγ*-KO macrophages were not (Figures 6b and c), suggesting that the relative increased TNF-*α* expression in *PPARγ*-KO macrophages was because of the failure of ATs phagocytosis. For further demonstration, the actin-filament polymerization-blocking agent cytochalasin B was used to inhibit macrophage phagocytic activity and the added ATs were found to have no effect on TNF-*α* expression in both macrophages strains (Figures 6b and c). Furthermore, the direct measurement of ATs engulfment by flow cytometry clearly showed that the phagocytic activity of *PPARγ*-KO macrophage was severely impaired (Figure 6d). These *in vitro* results suggested that *PPARγ*-KO macrophages produced excessive TNF-*α* because of an impaired phagocytic activity.

For *in vivo* study, TUNEL staining was applied to detect apoptotic cell accumulation in skin wound. We observed that the wounds (days 3, 5 and 7) of *PPARγ*-KO mice contained higher number of apoptotic cells compared with that in *PPARγ*-WT mice (Figure 6e and Supplementary Figure 6a). Furthermore, wound cells of *PPARγ*-KO mice had higher percentage of apoptotic neutrophils compared with *PPARγ*-WT mice (Figure 6f), indicating decreased apoptotic cell engulfment by *PPARγ*-KO macrophages in skin wound.

Shortly, these results indicated that PPAR*γ* deficiency impaired macrophage phagocytosis of apoptotic cells, resulting in macrophage producing of excessive TNF-*α*, contributing to increased TNF-*α* level in wounds.

### PPAR*γ* regulates the expression of genes involved in the phagocytosis of apoptotic cells by macrophages

To understand the underlying molecular mechanism of the deficit in apoptotic cell clearance observed in our mouse model, we profiled the transcription of a set of genes encoding phagocytosis-associated receptors and opsonins in *PPARγ*-WT and *PPARγ*-KO macrophages. Among them, Cd36 and Mertk are cell surface receptors and required for proper binding and consequent internalization of apoptotic cells.^39^ Mfge8, Gas6, and the C1q subunits C1qa, C1qb and C1qc are opsonins, which bind to apoptotic cell surface to initiate phagocytosis.^40^

**Figure 7:**
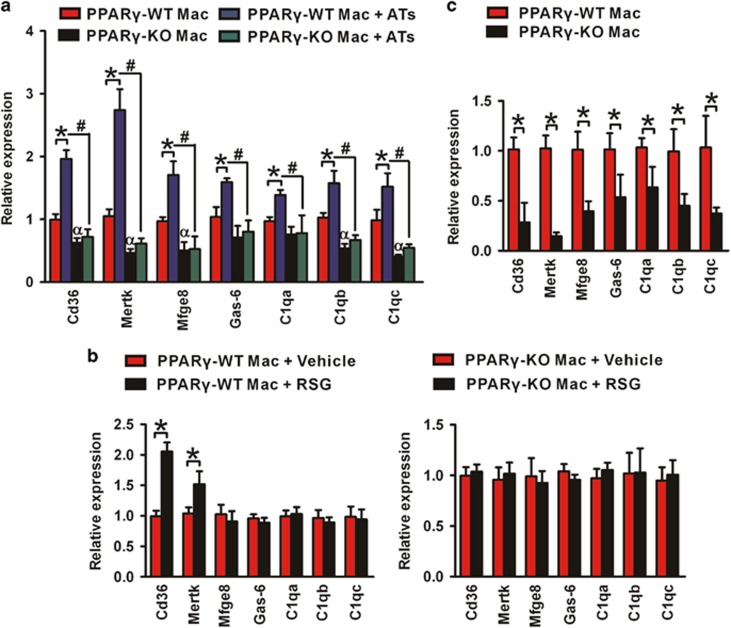
PPAR*γ* regulate the expression of macrophages receptors and opsonins involved in the engulfment of apoptotic cells. (**a**) mRNA expression of phagocytosis-related genes in peritoneal macrophages not added ATs or added ATs. (**b**) mRNA expression of phagocytosis-related genes in peritoneal macrophages treated with vehicle (ethanol) or RSG. (**c**) mRNA expression of phagocytosis-related genes in wound macrophages derived from PVA sponges after 5 days implantation. *P<0.05. ^#^*P*<0.05. ^*α*^*P*<0.05, *PPARγ*-KO macrophages *versus PPARγ*-WT macrophages. mRNA expression is normalized by *β*-actin, and gene expression is represented as fold change compared with *PPARγ*-WT macrophages, or *PPARγ*-WT or *PPARγ*-KO macrophages treated with Vehicle. Results are expressed as mean±S.D., *n*=3 for each group

In *PPARγ*-WT and *PPARγ*-KO macrophages, the expression of Cd36, Mertk, Mfge8, C1qb and C1qc was reduced in *PPARγ*-KO macrophages. However, PPAR*γ* deficiency had no effect on the expression of Gas6 and C1qa (Figure 7a). As digestion of apoptotic cells by macrophage leading to accumulation of cellular components, such as cholesterol and fatty acids, which may act as endogenous ligands for PPAR*γ*,^41^ we incubated both strains of macrophage with ATs, the results showed the mRNA levels of Cd36, Mertk, Mfge8, Gas6 and all three C1q subunits were higher in *PPARγ*-WT macrophages compared with the absence of ATs, which was not observed in *PPARγ*-KO macrophages (Figure 7a). Furthermore, activation of PPAR*γ* using rosiglitazone (RSG), which is a selective agonist of PPAR*γ*, increased the expression of Cd36 and Mertk in *PPARγ*-WT macrophages but not in *PPARγ*-KO cells. However, RSG did not significantly increase the expression of Mfge8, Gas6, C1qa, C1qb and C1qc in *PPARγ*-WT macrophages (Figure 7b). In addition, isolated macrophages from wound of *PPARγ*-KO mice decreased the expression of all the detected phagocytosis-related genes (Figure 7c). These results indicated that PPAR*γ* deficiency in macrophages decreased the expression of phagocytosis-related molecules, which may contribute to the reduced apoptotic cell clearance.

### Therapeutic targeting PPAR*γ* accelerates skin wound healing in normal mice

**Figure 8:**
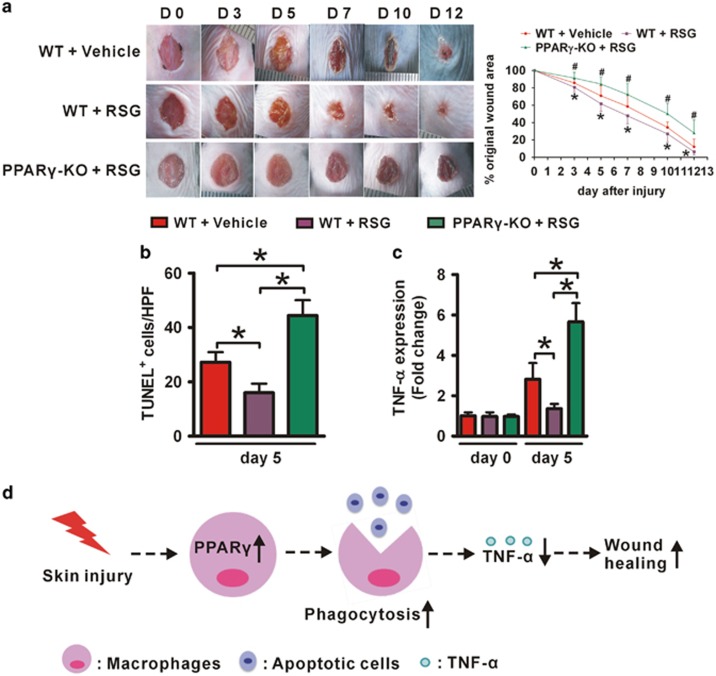
PPAR*γ* agonist accelerates wound healing in *WT* mice and suppresses apoptotic cell aggregation and TNF-*α* production in wounds, but not in *PPARγ*-KO mice. (**a**) Representative wounds, and statistical analysis of wound areas expressed as percentage of the initial (day 0) wound size. *n*=12, for each time point and group. *P<0.05, RSG-treated WT mice *versus* vehicle-treated WT mice; ^*#*^*P*<0.05, RSG-treated *PPARγ*-KO mice *versus* vehicle-treated WT mice. (**b**) Quantification of TUNEL^+^ cells in 5-day-old wounds in 3–5 high-power fields (HPFs) per mice. (**c**) mRNA expression of TNF-*α* in normal skin and 5-day-old wounds. Both (**b** and **c**), *n*=3 for each time point and group. All data are expressed as mean±S.D. *P<0.05. (**d**) After tissue injury, wound macrophage PPAR*γ* is upregulated to timely disposal of apoptotic cells, resulting in decreased local TNF-*α* expression to enhancing wound healing

Owing to PPAR*γ* upexpression in wounded skin and delayed wound healing in mice with macrophage PPAR*γ* deficiency, we speculated that PPAR*γ* may have a vital role in regulating skin wound healing. RSG treatment of skin wound in WT mice resulted in accelerated wound healing, reduced apoptotic cell aggregation and lower local TNF-*α* expression compared with a control treatment (Figures 8a–c and Supplementary Figure 6b). However, RSG has no therapeutic effects in *PPARγ*-KO mice (Figures 8a–c and Supplementary Figure 6b).

## Discussion

PPAR*γ* has a key role in regulating macrophage phagocytic activity during skin wound healing. In this study, PPAR*γ* was upregulated in wounded skin and wound macrophages indicating that macrophage PPAR*γ* may regulate the healing process. Thus, we generated macrophage PPAR*γ* deficiency mice. These mice exhibited efficient and specific macrophages PPAR*γ* ablation, not targeting other immune cells, such as neutrophils, dendritic cells, T cells and B cells, which may influence the skin wound inflammation. Furthermore, the expression of PPAR*γ* in skin was not upregulated during wound healing in *PPARγ*-KO mice, suggesting an ablation of PPAR*γ* in wound macrophage. *PPARγ*-KO mice reduced granulation tissue formation, collagen deposition and angiogenesis, and delayed wound closure. In *PPARγ*-KO mice, the TNF-*α* expression was increased in skin wound and local inhibition of TNF-*α* rescued healing deficit in *PPARγ*-KO mice, indicating that the excessive TNF-*α* contributed to the impaired skin wound healing in *PPARγ*-KO mice. Next, we observed that *PPARγ*-KO wound macrophages released more TNF-*α* compared with their control macrophages. Furthermore, PPAR*γ* deficiency impaired macrophage phagocytosis of apoptotic cells through downregulating phagocytosis-related molecules in macrophage, resulting in excessive TNF-*α* expression by *PPARγ*-KO macrophages. Meanwhile, increased apoptotic cells accumulation in *PPARγ*-KO wounds was observed. These results directly or indirectly demonstrated that the impaired macrophage phagocytosis contributes to the excessive TNF-*α* in wound of *PPARγ*-KO mice. Therefore, macrophage PPAR*γ* deficiency decreased the expression of phagocytosis-related molecules, leading to deficit in clearance of apoptotic cells, resulting in impaired skin wound healing.

The timely and efficient clearance of apoptotic cells by macrophages is the key to skin wound healing.^11^ Macrophage phagocytosis of apoptotic cells prevents further tissue damage by protecting tissue from exposure to toxic components of dying cells, and suppresses the production of pro-inflammatory cytokines, and enhancing the expression of anti-inflammatory cytokines and growth factors, resulting in resolution of wound inflammation and promoting wound healing.^42, 43^ Deficit in macrophage phagocytosis function during wound healing are related with chronic, non-healing wounds.^13, 14, 15^ Our study provides the first evidence of PPAR*γ* regulating wound macrophage phagocytosis function during skin wound healing. In addition, PPAR*γ* deficiency resulted in impaired macrophage phagocytosis function, contributing to delayed wound closure, which is consistent with the importance of apoptotic cell clearance in wound healing. Although several studies have observed macrophage phagocytosis deficit in chronic, non-healing wounds,^13, 14, 15^ the regulatory mechanisms underlying wound macrophage phagocytosis function are not fully deciphered. Differ from adhesion molecule *β*_2_ integrins, which mediate adhesion-dependent phagocytosis of apoptotic cells by wound macrophages,^28, 31^ PPAR*γ*, as a nuclear transcription factor, transcriptionally regulates wound macrophage phagocytosis function. This finding may explain the poor wound healing in patients with chronic granulomatous disease, because macrophages from mice with chronic granulomatous disease have low levels of PPAR*γ* expression,^44^ or poor wound healing in patients with other diseases present macrophage PPAR*γ* low expression, and future studies are sure to test them.

Among all the tested macrophages phagocytosis-related molecules in our investigation, PPAR*γ* consistently affected the expression of Cd36 and Mertk in *PPARγ*-KO macrophages. Although Cd36 is well known to be upregulated by PPAR*γ* activation, the regulation of Mertk by PPAR*γ* has been reported.^45, 46^ However, the expression of Mertk is induced by activating liver X receptor (LXR),^47^ and activating PPAR*γ* will upregulate LXR,^48^ suggesting that Mertk expression may be induced by PPAR*γ* via LXR.

TNF-*α* is a key factor impacting the healing process.^35, 37^ Excessive TNF-*α* expression in wound impaired the functions of keratinocytes, fibroblasts and endothelial cells,^37, 49, 50^ resulting in reduced reepithelialization, collagen deposition, angiogenesis and granulation tissue formation, and severely delayed wound healing.^34, 36^ Our results showed wound *PPARγ*-KO macrophages released excessive TNF-*α*, contributing to higher TNF-*α* expression in wounds, resulting in impaired wound healing in *PPARγ*-KO mice. Although in chronic, non-healing wounds, high expression of TNF-*α* by wound macrophages were observed, the underlying mechanisms are not fully known. However, the phagocytosis of apoptotic cells is associated with an anti-inflammatory response, such as suppressing TNF-*α* expression, in macrophages and the failure of apoptotic cell clearance is associated with chronic wound, suggesting the involvement of apoptotic cell engulfment in TNF-*α* expression regulation in skin wound. Here, we revealed that PPAR*γ* regulates skin wound macrophages TNF-*α* expression by orchestrating their phagocytic function. It is well established that apoptotic cells are digested and cellular components, such as cholesterol and fatty acids, are released after engulfed by macrophages. These cellular components are ligands for PPAR*γ*, which may induce PPAR*γ* sumoylation to attenuate the removal of nuclear receptor corepressor from the *κ*B site within the TNF-*α* promoter, resulting in blocking the transactivation of NF-*κ*B and reducing TNF-*α* production in macrophages.^51^ Therefore, PPAR*γ* can directly contribute to the TNF-*α* expression regulation induced by apoptotic cell engulfment. Correspondingly, the increased engulfment of apoptotic cells may enhance PPAR*γ* activation to suppress TNF-*α* production. Our data here showed that PPAR*γ* enhanced phagocytosis of apoptotic cells through increasing the expression of macrophage phagocytosis-related molecules, especially CD36 and Mertk, suggesting that PPAR*γ* may also indirectly reduce TNF-*α* expression via upregulation of CD36 and Mertk.

Although our study here clearly showed that PPAR*γ* was important for regulating wound macrophage phagocytosis function, the contribution of PPAR*γ* to skin wound healing remains controversial. Previous studies showed that in fibroblasts, PPAR*γ* agonists block TGF-*β*-induced *α*SMA, which is a myofibroblast differentiation marker and collagen expression,^52, 53^ and deletion of PPAR*γ* in fibroblasts enhances dermal wound closure, indicating that PPAR*γ* acts in fibroblasts retarding tissue repair. However, our results showed that PPAR*γ* upregulated in wounded skin, which is consistent with previous study^54^ and in wound macrophages, and mice with macrophage PPAR*γ* deficiency severely impaired wound healing, suggesting that PPAR*γ* has a regulatory role in wound healing and acts in macrophages enhancing the healing process. Despite the controversial roles of PPAR*γ* in macrophages and fibroblasts during wound healing, our data showed that PPAR*γ* agonist accelerated the wound healing in WT but not *PPARγ*-KO mice, suggesting that PPAR*γ* agonist accelerated wound healing because of activating macrophages PPAR*γ*. So we concluded that activating PPAR*γ* is helpful for improving skin wound healing.

In summary, our studies elucidated the pathophysiological mechanism of macrophage PPAR*γ* in skin wound healing. After tissue injury, macrophage PPAR*γ* is upregulated to promote timely disposal of apoptotic cells through increased the phagocytosis-related molecules expression, contributing to decreased local TNF-*α* expression to enhancing tissue repair (Figure 8d). Furthermore, we showed that activating PPAR*γ* accelerated normal wound healing, providing the evidence of PPAR*γ* may be a candidate for treatment of wound healing.

## Materials and Methods

### Mice

C57BL/6J male mice (8–12 weeks) were purchased from the Animal Center, Research Institute of Surgery and Daping hospital, TMMU, Chongqing, China. Mice carrying floxed alleles of *PPARγ* (*PPARγ*^*f/f*^) and mice bearing the *lysozyme-M Cre* (*LysMCre*) recombinase transgene were purchased from the Jackson Laboratory (Mount Desert Island, ME, USA). *PPARγ*^*f/f*^ mice and *LysMCre* mice were crossed to generate offspring with macrophage-specific deficiency of the *PPARγ* gene. The *PPARγ*^*f/f*^*LysMCre*^*−*^ mice (*PPARγ*-WT) were used as WT control as previous reports and *PPARγ*^*f/f*^*LysMCre*^*+*^ mice are referred to as *PPARγ*-KO mice.^22, 55^ All mice were on a C57BL/6 background and housed under a 12-h light–12-h dark cycle with free access to food and water. Wound-healing experiments were performed with male mice between 8 and 12 weeks of age. All animal procedures were in accordance with a protocol approved by the Local Administration District Official Committee. All efforts were made to minimize the number of animals and their suffering.

### Analyses of macrophage-specific PPAR*γ* deficient mice

The genotyping of determining *PPARγ*-WT and *PPARγ*-KO mice was performed by PCR of DNA obtained from tail biopsies. Primary peritoneal macrophages were recovered from mice injected with 3% thyoglicolate for 3 days. The mRNA levels and protein expression of PPAR*γ* in both macrophage strains were performed by RT-PCR and WB. PPAR*γ* expression in isolated wound macrophages and neutrophils, and splenic T cells, B cells and dendritic cells were analyzed by flow cytometry.

### Wound-healing model

Full-thickness (including the *panniculus carnosus*) wounds were created in the dorsal skin under sterile conditions. Briefly, mice were anesthetized with peritoneal injection (i.p.) with pentobarbital sodium (50 mg/kg; Boster, Wuhan, China). After depilation with 8% Na_2_S and cleaning with povidone-iodine and 75% ethanol, the dorsal skin was picked up at the midline and punched with a sterile disposable biopsy punch (6 mm in diameter; Miltex, New York, NY, USA), generating one wound on the midline or two wounds on each side of the midline. One or four wounds per mice were induced. At indicated time points, each wound was digitally photographed, and wound areas were quantified using CorelDRAW 9 (Corel Software, Oakland, CA, USA). Changes in wound areas over time were expressed as the percentage of initial (day 0) wound area.

### PVA sponges implantation and wound cell isolation

Mice were anesthetized with pentobarbital (50 mg/kg i.p.) and dorsal skin was depilated. Six PVA sponges (unlimited), measuring 1 × 1 × 0.6 cm, were inserted into individual s.c. pockets through a midline dorsal incision under sterile condition, and the skin was closed with clips.^56^ At specified times, mice were killed by CO_2_ asphyxiation, and the sponges were removed and a single-wound cell suspension was generated from sponges by repeated compression. The cell suspension was filtered through a 70 *μ*m nylon cell strainer (Falcon, Shanghai, China) to remove all the sponge debris. When necessary, cells were subjected to red cell lysis buffer (Invitrogen, San Diego, CA, USA), followed by reconstitution in PBS. The s.c. sponge model is extensively used for wound-healing studies especially those investigating wound inflammation.^57, 58, 59, 60^ The primary difference between s.c. sponge model and excisional wound model is low-grade bacterial contamination of open wounds.^61^ Therefore, we routinely checked bacterial contamination in our excisional wounds using protocols described previously.^62^ As previous studies reported, although low-grade contamination was observed in superficial tissues, deep-tissue biopsies had not shown any bacterial contamination, suggesting that excisional deep-tissue wound cells are similar to that derived from PVA sponge.^62^

### Total RNA isolation and quantitative RT-PCR

Total RNA was isolated using Tripure Isolation Reagent (Roche Group, Shanghai, China) and reverse transcribed into cDNA using Quantscript RT Kit (Tiangen Biotech, Beijing, China). The resulting cDNA was used to measure quantitatively the expression of genes using Quant Reverse Transcriptase according to the manufacturer's protocol (Tiangen Biotech, Beijing, China). Real-time measurements of gene expression were performed with an iCycler thermocycler system and iQ5 optical system software (Bio-Rad Laboratory, Richmond, CA, USA). All primers were synthesized by Invitrogen Technologies (Carlsbad, CA, USA) and primer sequences used are presented as follows: *β*-actin (forward, 5′-TGGAATCCTGTGGCATCCATGAAA-3′ reverse, 5′-TAAAACGCAGCTCAGTAACAGTCCG-3′), PPAR*γ* (forward, 5′-TATCACTGGAGATCTCCGCCAACAGC-3′ reverse, 5′-GTCACGTTCTGACAGGACTGTGTGAC-3′), collagen type 1 (forward, 5′-ATCACTGCAAGAACAGCGTA-3′ reverse, 5′-TGTTTTCCAAAGTCCATGTG-3′), VEGF (forward, 5′-TGGATGTCTACCAGCGAAGC-3′ reverse, 5′-ACAAGGCTCACAGTGATTTT-3′), TNF-*α* (forward, 5′-AACTAGTGGTGCCAGCCGAT-3′ reverse, 5′-CTTCACAGAGCAATGACTCC-3′), Cd36 (forward, 5′-TCGGAACTGTGGGCTCATTG-3′ reverse, 5′-CCTCGGGGTCCTGAGTTATATTTTC-3′), Mertk (forward, 5′-GTGGCAGTGAAGACCATGAAGTTG-3′ reverse, 5′-GAACTCCGGGATAGGGAGTCAT-3′), Gas6 (forward, 5′-TCTTCTCACACTGTGCTGTTGCG-3′ reverse, 5′-GGTCAGGCAAGTTCTGAACACAT-3′), Mfge8 (forward, 5′-GGACATCTTCACCGAATACATCTGC-3′ reverse, 5′-TGATACCCGCATCTTCCGCAG-3′), C1qa (forward, 5′-AAAGGCAATCCAGGCAATATCA-3′ reverse, 5′-TGGTTCTGGTATGGACTCTCC-3′), C1qb (forward, 5′-AACGCGAACGAGAACTATGA-3′ reverse, 5′-ACGAGATTCACACACACAGGTTG-3′) and C1qc (forward, 5′-CAACGCCCTCGTCAGGTT-3′ reverse, 5′-ACAACCCAAGCACAGGGAAGT-3′). The RT-PCR data were analyzed by the method of 2^*−*ΔΔCt^ and the data were normalized to *β*-actin.

### Western blotting

Wound tissues and cultured cells were homogenized in ice-cold RIPA with 1 mg/ml of a protease inhibitor cocktail (Beyotime, Shanghai, China). The homogenates were centrifuged at 14 000  r.p.m. for 5 min at 4 °C and supernatants were collected. Subsequently, BCA assays were performed to determine the protein concentration of the supernatants. Standard protocol was used and following antibodies were applied: PPAR*γ* (Abcam Inc., San Francisco, CA, USA), TNF-*α* (Santa Cruz Biotechnology, Santa Cruz, CA, USA), GAPDH (Goodhere Biotechnology, Hangzhou, China), the goat anti-rabbit IgG-HRP secondary antibody (Novus Biologicals, Littleton, CO, USA) and the goat anti-mouse IgG-HRP secondary antibody (Beyotime). The blots were detected by chemiluminescence with enhanced chemiluminescence reagent (BeyoECL Plus, Beyotime).

### Histology

Wounded tissues were harvested, fixed in 4% paraformaldehyde, dehydrated, bisected, mounted in paraffin and sectioned for H&E staining, Masson trichrome staining and immunohistochemistry. Granulation tissues were stained using H&E. Collagen was stained with Masson trichrome. Immunohistochemistry was carried out with following antibodies: PPAR*γ* (Abcam Inc.), F4/80 (Abcam Inc.), Ly-6G (eBioscience, San Diego, CA, USA), CD31 (Boster), the goat anti-rat IgG-biotin secondary antibody (Invitrogen) and the goat anti-rabbit IgG-biotin secondary antibody (Invitrogen). The protocol used for immunohistochemistry was performed as reported previously.^63^ Briefly, paraffin-embedded tissue blocks were cut into 4 *μ*m sections. After the sections were dewaxed and rehydrated, antigen retrieval was performed by microwaving in citrate buffer (pH 6.0). The sections were cooled to room temperature (RT), and endogenous peroxidase activity was blocked by incubation with 3% hydrogen peroxidase (H_2_O_2_) for 10 min. The sections were incubated with 3% albumin boving V to block nonspecific binding at RT. Thereafter, the sections were incubated with primary antibodies overnight at 4 °C. After washing, the sections were incubated with corresponding secondary antibodies for 30 min at RT. Subsequently, the Vecta-stain ABC kit (Vector Laboratories, San Diego, CA, USA) was used for the avidin–biotin complex method according the manufacturer's instruction. Peroxidase activity was visualized with DAB Elite kit (K3465, DAKO, Copenhagen, Denmark). The sections were lightly counterstained with hematoxylin and dehydrated through an ethanol series to xylene and mounted.

Tunel staining was performed using a commercially available kit (Derma TACS, Trevigen Inc., Gaithersburg, MD, USA).

All the sections were viewed using a light and fluorescence microscope. For image quantification, 3–5 high-powered images were quantified for each data point per animal.

### Local TNF-*α* restoration and PPAR*γ* agonist treatment

In TNF-*α* restoration experiments, wound margins were s.c. injected with 10 *μ*g per wound purified aTNF-*α* (Sungene, Tianjin, China) on days 3, 4, 5, 6 and 7 after wounding. Mice treated with isotype control antibody (Sungene) served as mock controls. For PPAR*γ* agonist RSG (Sigma Aldrich, St. Louis, MO, USA) treatment, mice were given RSG (10 mg/kg) or vehicle (carboxymethyl cellulose) (Sangon Biotech, Shanghai, China) via oral gavage for 2 days before wounding and every day thereafter until day 12 after wounding.

### Gene expression and cytokine secretion assays

Primary peritoneal macrophages were collected from abdomen of mice, which were injected with 3% thyoglicolate for 3 days. Cells were incubated in RPMI 1640 medium supplemented with 10% FCS. To generate ATs, thymi from 3- to 4- week-old C57BL/6 mice were harvested and ground, filtered, pelleted and resuspended in RPMI 1640 medium supplemented with 10% FCS. Apoptosis was induced by treatment with 1 *μ*M of dexamethasone (Sigma Aldrich) for 6 h. For AT assays, ATs were added to macrophages (5 : 1) for 60 min or 24 h. When needed, ATs were removed by gently washing, and macrophages were incubated with 100 ng/ml LPS for additional 5 h for gene expression analysis and 18 h for cytokine secretion studies. For ligand assays, macrophages were incubated with 100 nM RSG for 24 h. Gene expression was performed by RT-PCR and cytokine secretion was quantified in culture supernatants using commercially available ELISA kits (BD Biosciences, Franklin Lakes, NJ, USA).

### *In vitro* phagocytosis assays

CFSE-labeled ATs were added to peritoneal macrophages in a 5 : 1 ratio and co-cultured in RPMI supplemented with 10% FBS at 37 °C for 60 min. After incubation with ATs, macrophages were gently washed several times with cold PBS and Cell Dissociation Buffer, Enzyme Free PBS-based (Invitrogen). A single-cell suspension of macrophages was stained with PE-conjugated anti-mouse F4/80 antibody (Sungene Biotech, Tianjin, China) and cells were analyzed by flow cytometry.

### Flow cytometric analyses of wound cells isolated from PVA sponges

Wound cells were isolated from PVA sponges at indicated time points. Antibodies used for cells staining included: Ly-6G, F4/80, CD11b, annexin V, anti-rabbit IgG (FITC-conjugated secondary antibody) all from Sungene Biotech; PPAR*γ* from Abcam Inc.; TNF-*α* and normal rabbit IgG from Santa Cruz Biotechnology. Cells were analyzed with a FACScan (BD Biosciences). Flow cytometry analysis was done with the FlowJo software (FlowJo, Hangzhou, China).

### Flow cytometric analyses of splenic T cells, B cells and dendritic cells

The spleen was cut from abdomen and ground, and erythrocytes were lysed with red cell lysis buffer. The cells were then resuspended in PBS. Antibodies used for cells staining included: CD3, CD4, CD8, CD19, CD11b, CD11c, anti-rabbit IgG (FITC-conjugated secondary antibody) all from Sungene Biotech; PPAR*γ* from Abcam Inc.; normal rabbit IgG from Santa Cruz Biotechnology. Cells were analyzed with a FACScan. Flow cytometry analysis was done with the FlowJo software.

### Statistical analysis

Data were statistically analyzed using GraphPadPrism 5.0 (GraphPad Software, Beijing, China) and expressed as the mean±S.D. for the indicated number of independently performed duplicated experiments. Statistical significance was determined by two-tailed Student's *t-*test. For all statistical analyses, significance levels were set at *P-*values <0.05.

## Abbreviations

ATs: apoptotic thymocytes
KO: knock out
LPS: lipopolysaccharide
PPAR*γ*: peroxisome proliferator-activated receptor gamma
RSG: rosiglitazone
TNF-*α*: tumor necrosis factor alpha
WT: wild type
