# Supplementary figures and images for: Macrophage peroxisome proliferator-activated receptor γ deficiency delays skin wound healing through impairing apoptotic cell clearance in mice
Source: Cell Death Dis. 2015 Jan 15;6(1):e1597–. doi: 10.1038/cddis.2014.544 (PMC4669743; doi:10.1038/cddis.2014.544)

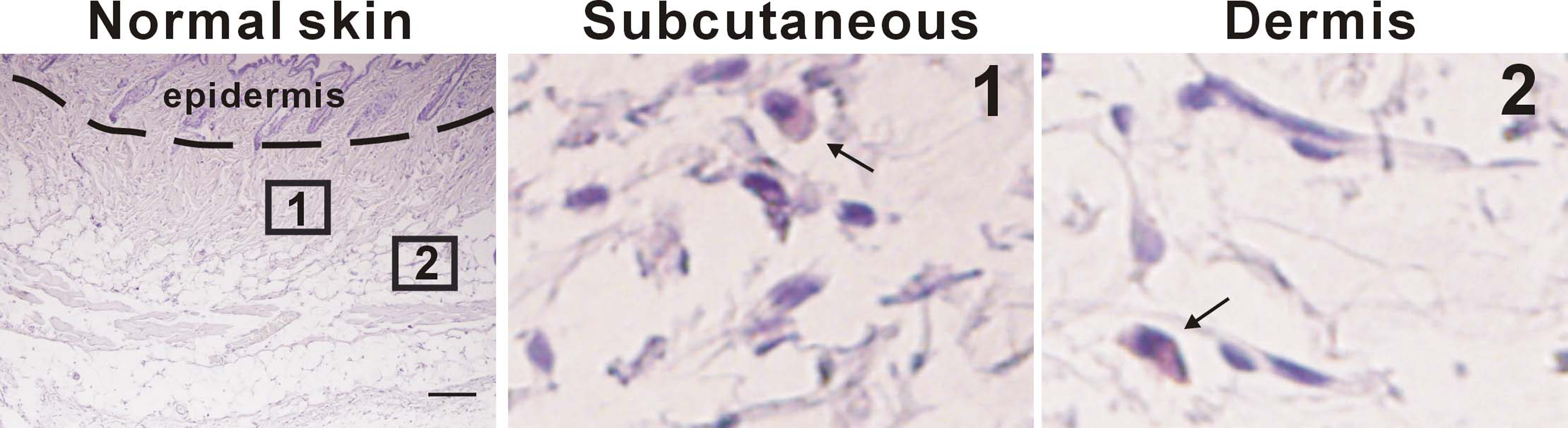

Supplement: Supplementary Figure 1 [file cddis2014544x1.tif]

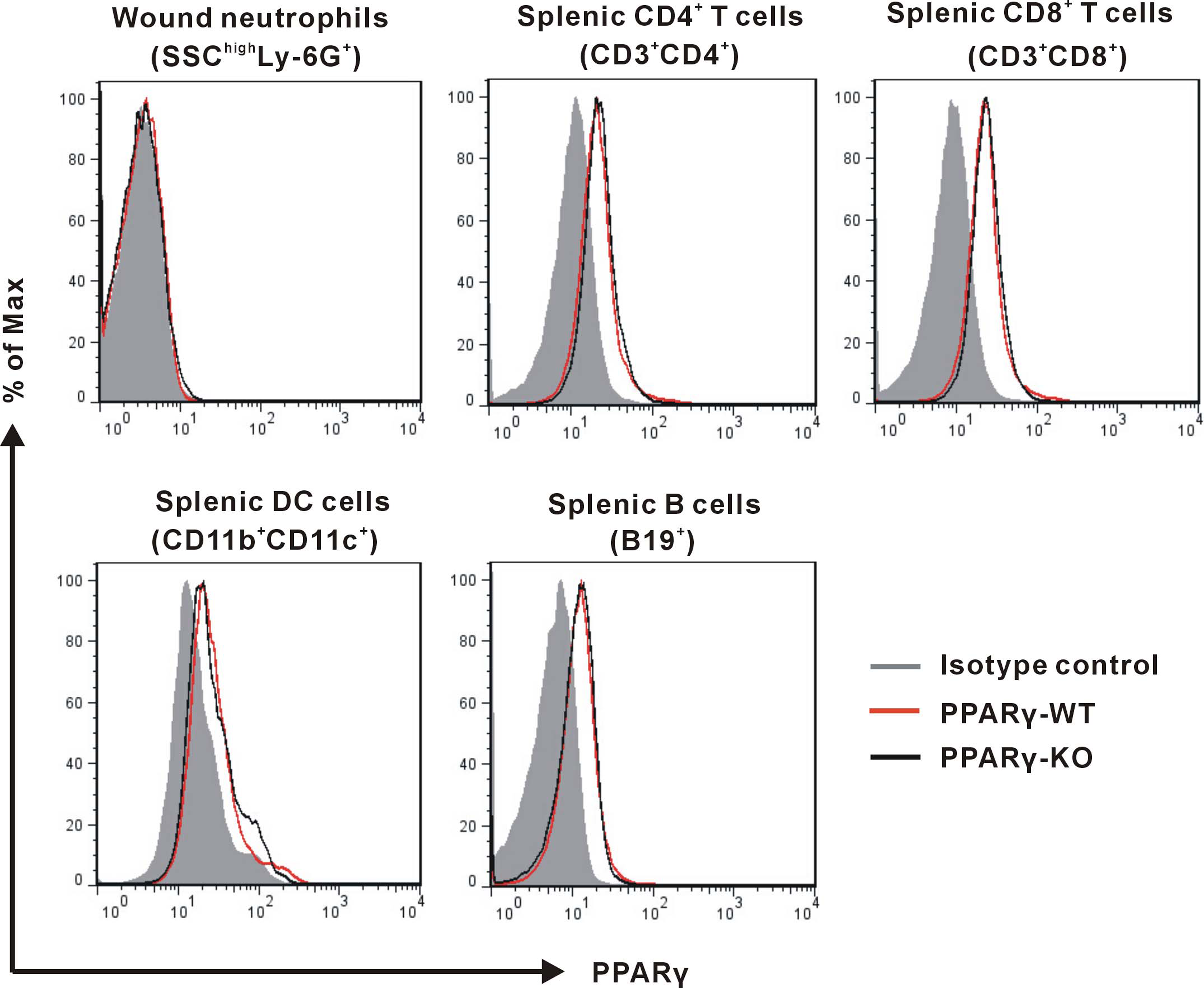

Supplement: Supplementary Figure 2 [file cddis2014544x2.tif]

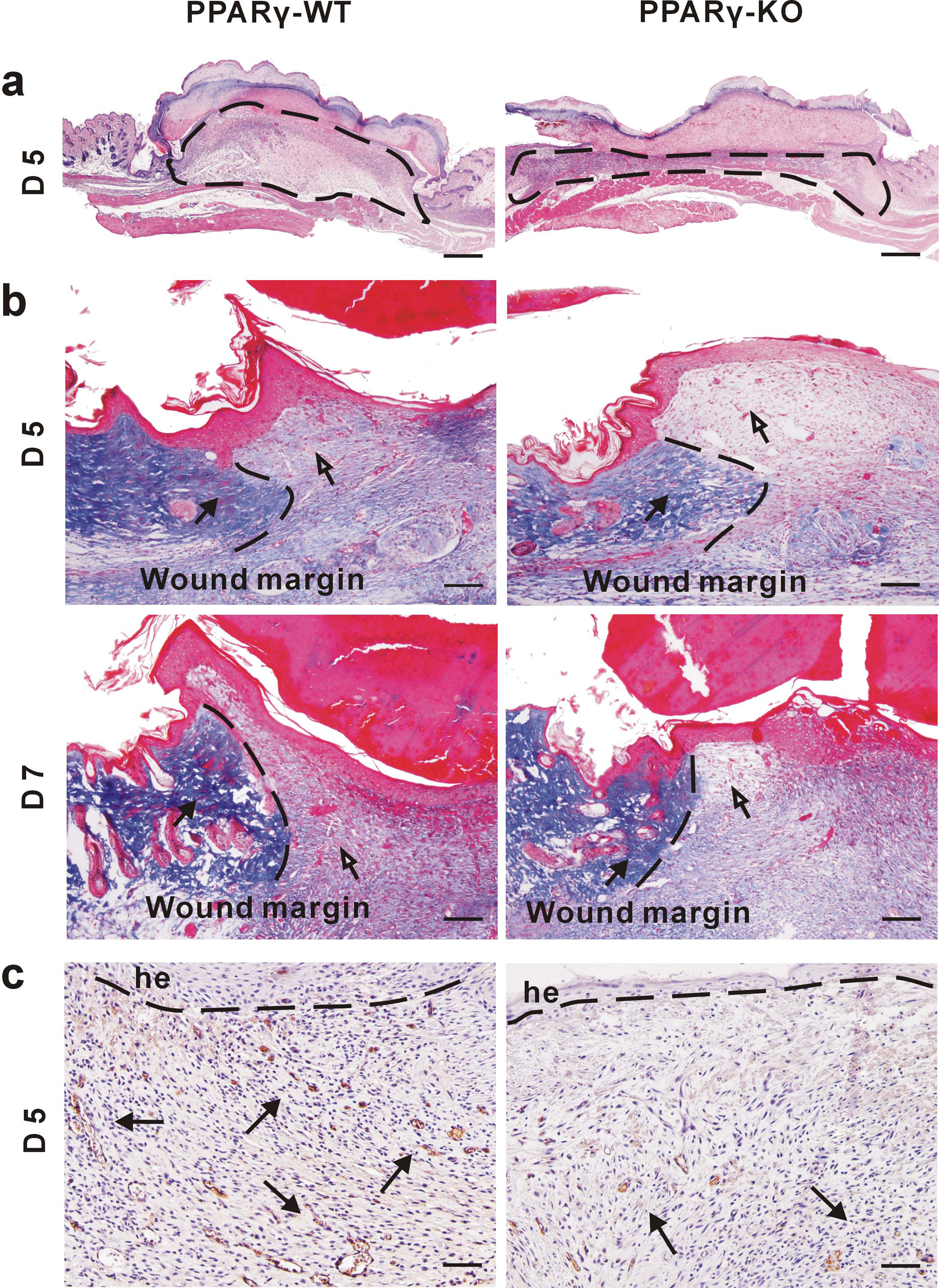

Supplement: Supplementary Figure 3 [file cddis2014544x3.tif]

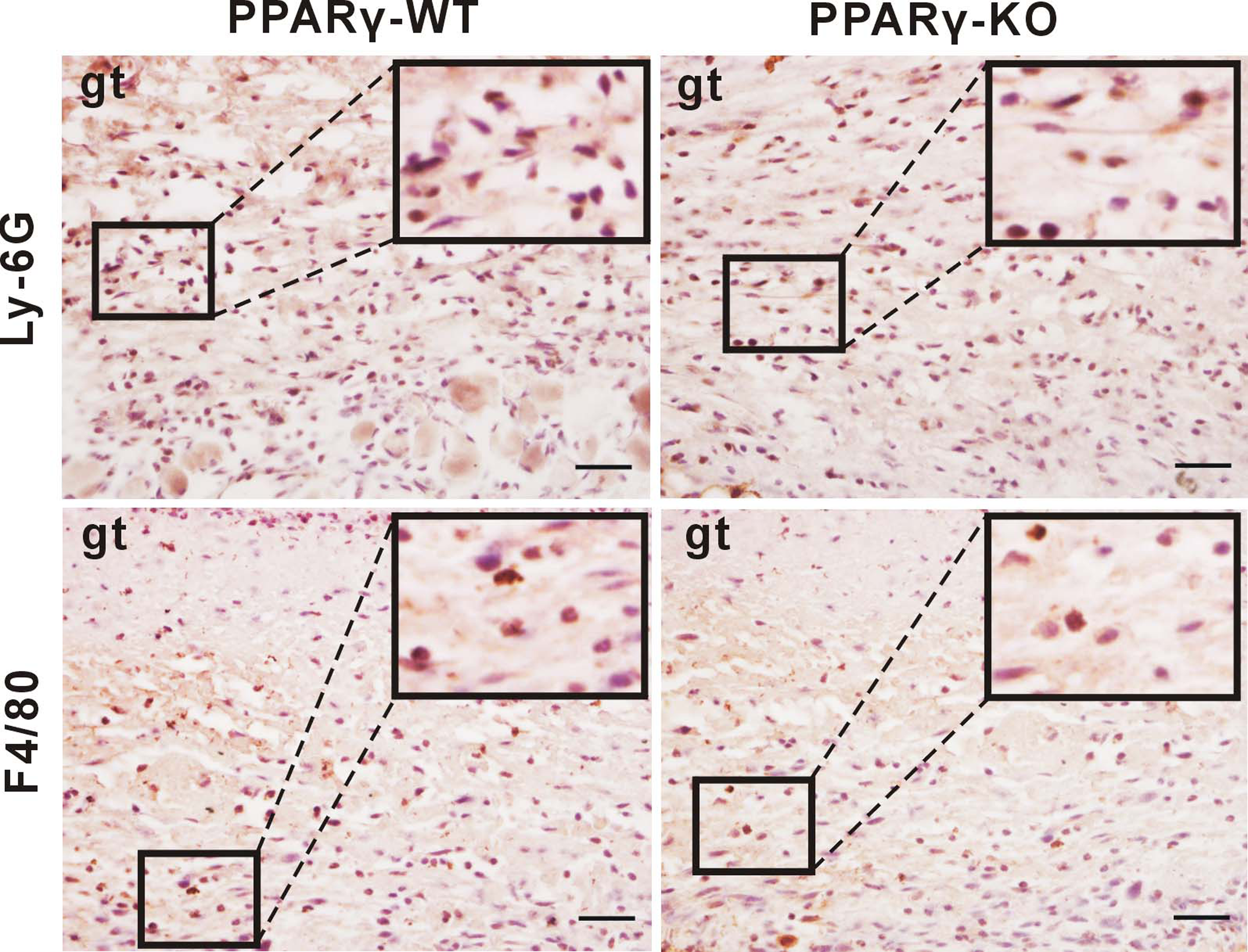

Supplement: Supplementary Figure 4 [file cddis2014544x4.tif]

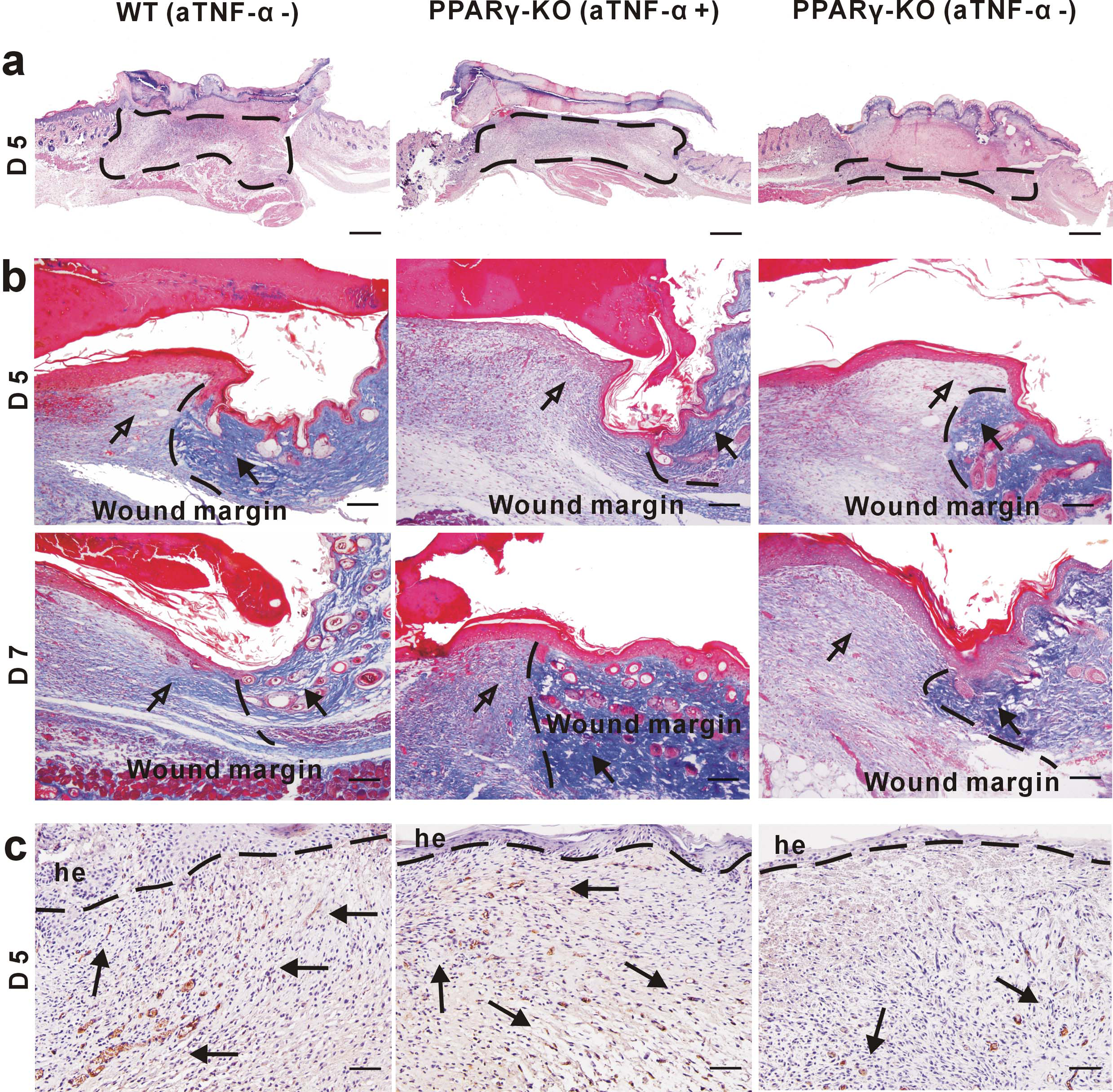

Supplement: Supplementary Figure 5 [file cddis2014544x5.tif]

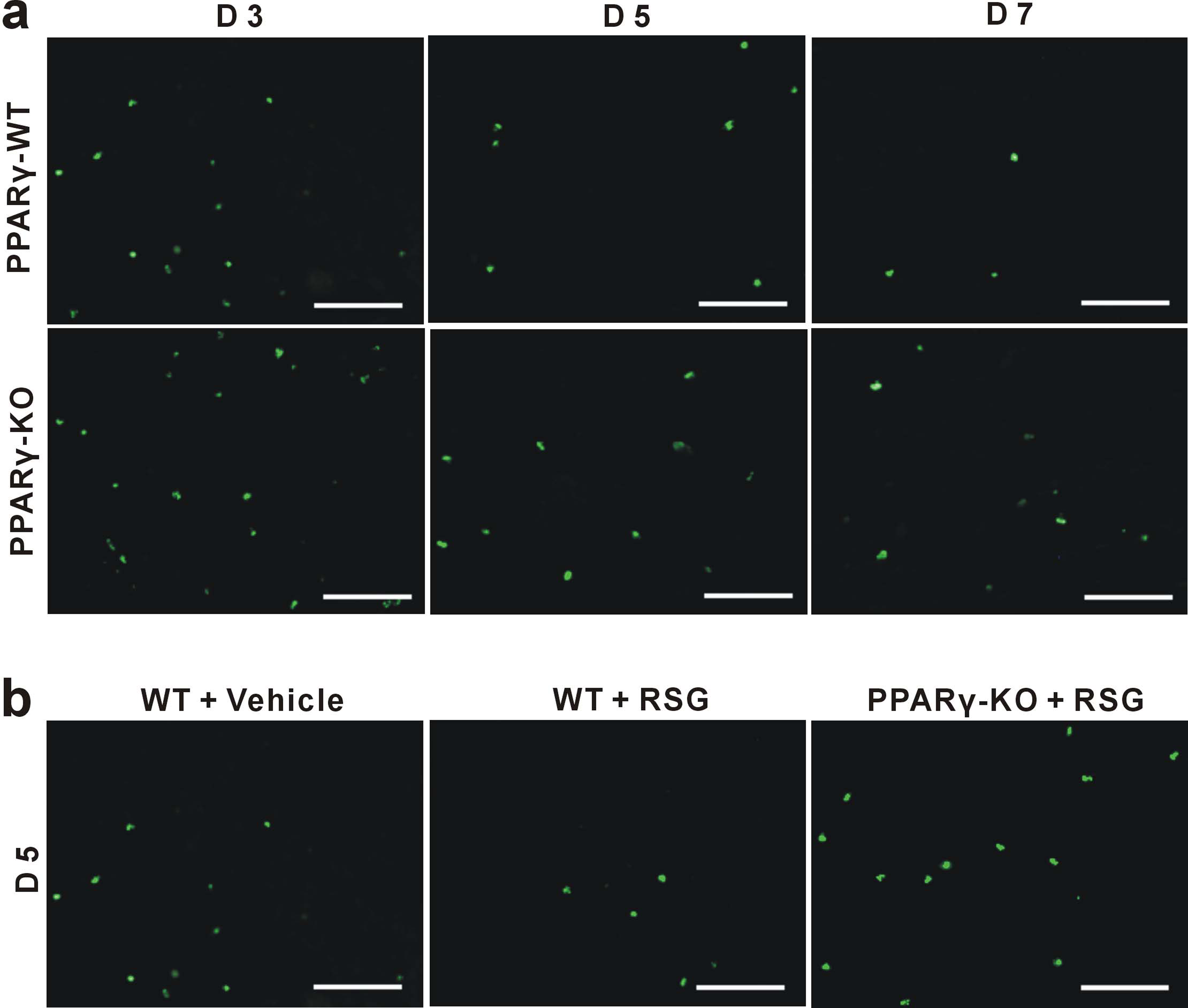

Supplement: Supplementary Figure 6 [file cddis2014544x6.tif]
