# Supplementary material for: Macrophage peroxisome proliferator-activated receptor γ deficiency delays skin wound healing through impairing apoptotic cell clearance in mice
Source: Cell Death Dis. 2015 Jan 15;6(1):e1597–. doi: 10.1038/cddis.2014.544 (PMC4669743; doi:10.1038/cddis.2014.544)
Supplement: Supplementary Figure Legends [file cddis2014544x7.doc]

**Supplementary Figure legends**

**Figure S1. PPARγ expression in normal skin of WT mice.** Immunohistochemical staining for PPARγ in normal skin. Boxed areas of subcutaneous (number 1) and dermis (number 2) tissue in the left panel are enlarged in the middle and right panel; black closed arrows: PPARγ^+^ cells. Black hatched line outlines epidermis. Scale bar = 50 μm. Images are representative, n = 3.

**Figure S2. PPARγ expression in wound neutrophils, and splenic T cells, B cells and dendritic cells.** Flow cytometric analysis of PPARγ expression in wound neutrophils, and splenic T cells, B cells and dendritic cells of *PPARγ-*WT and *PPARγ-*KO mice. Isotype control, gray histogram; PPARγ, unshaded histogram (red histogram: *PPARγ-*WT; black histogram: *PPARγ-*KO). Images are representative, n = 3 for each group.

**Figure S3. Granulation tissue formation, collagen deposition and angiogenesis in *PPARγ-*WT and *PPARγ-*KO wounds.** (a) HE staining for granulation tissue in 5-day-old wounds. Black hatched line outlines granulation tissue, scale bar = 500 μm. (b) Masson staining for 5- and 7-day-old wounds. Black hatched line outlines wound margin, closed arrow marks old collagen and open arrow marks newly formed collagen, scale bar = 50 μm. (c) CD31 staining for 5-day-old wounds. Black hatched line outlines hyperproliferative epidermis. he, hyperproliferative epidermis. Scale bar = 50 μm. Images are representative, n = 3 for each time point and group.

**Figure S4. Immunohistochemical staining for wound neutrophils and macrophages.** Wound neutrophils were stained with a neutrophil-specific antibody (Ly-6G), and wound macrophages were stained with a macrophage-specific antibody (F4/80) in 3-day-old wounds. The areas of granulation tissues were showed in images; gt, granulation tissue. Boxed areas are magnified, scale bar = 50 μm. Images are representative, n = 3 for each group.

**Figure S5. Granulation tissue formation, collagen deposition and angiogenesis in WT (aTNF-α -), *PPARγ-*KO (aTNF-α +) and *PPARγ-*KO (aTNF-α -) wounds.** (a) HE staining for granulation tissue in 5-day-old wounds. Black hatched line outlines granulation tissue, scale bar = 500 μm. (b) Masson staining for 5- and 7-day-old wounds. Black hatched line outlines wound margin, closed arrow marks old collagen and open arrow marks newly formed collagen, scale bar = 50 μm. (c) CD31 staining for 5-day-old wounds. Black hatched line outlines hyperproliferative epidermis. he, hyperproliferative epidermis. Scale bar = 50 μm. Images are representative, n = 3 for each time point and group.

**Figure S6. TUNEL staining for wound apoptotic cells.** TUNEL staining for 3-, 5- and 7-day-old wounds of *PPARγ-*WT mice and *PPARγ-*KO mice, and for 5-day-old wounds of vehicle-treated WT mice, RSG-treated WT mice and RSG-treated *PPARγ-*KO mice. Scale bar = 50μm. Images are representative, n = 3 for each time point and group.
